# Risk of Periodontitis in Patients with Gastroesophageal Reflux Disease: A Nationwide Retrospective Cohort Study

**DOI:** 10.3390/biomedicines10112980

**Published:** 2022-11-19

**Authors:** Xin Li, Hitesh Singh Chaouhan, Yao-Ming Wang, I-Kuan Wang, Cheng-Li Lin, Te-Chun Shen, Chi-Yuan Li, Kuo-Ting Sun

**Affiliations:** 1 Graduate Institute of Biomedical Sciences, China Medical University, Taichung 40402, Taiwan; 2 Department of Radiology, Taichung Tzu Chi Hospital, Buddhist Tzu Chi Medical Foundation, Taichung 40402, Taiwan; 3 Department of Internal Medicine, China Medical University Hospital, Taichung 40402, Taiwan; 4 School of Medicine, China Medical University, Taichung 40402, Taiwan; 5 Management Office for Health Data, China Medical University Hospital, Taichung 40402, Taiwan; 6 Department of Anesthesiology, China Medical University Hospital, Taichung 40402, Taiwan; 7 Department of Pediatric Dentistry, China Medical University Hospital, Taichung 40402, Taiwan; 8 School of Dentistry, China Medical University, Taichung 40402, Taiwan

**Keywords:** gastroesophageal reflux, periodontitis, risk factors, cohort studies

## Abstract

Background: Gastroesophageal reflux disease (GERD) is the most common digestive clinical problem worldwide that affects approximately 20% of the adult populations in Western countries. Poor oral hygiene has been reported to be associated with GERD as an atypical clinical complication. However, evidence showing the relationship between GERD and the risk of periodontitis is less clear. The present study aimed to use a retrospective cohort study design to further clarify the association between GERD and the subsequent risk of periodontitis. Methods: The risk of periodontitis in patients with GERD was investigated by analyzing epidemiological data from the Taiwan National Health Insurance Research Database from 2008 to 2018. We selected 20,125 participants with a minimum age of 40 years in the GERD group and 1:1 propensity-matched these with non-GERD individuals by sex, age, and comorbidities. The incidence of periodontitis was determined at the end of 2018. A Cox proportional hazards regression model was used to evaluate the risk of periodontitis in patients with GERD. Results: The overall incidence rate of the periodontitis risk was 1.38-fold higher (30.0 vs. 21.7/1000 person years, adjusted hazard ratio (aHR) = 1.36, 95% confidence interval (CI) = 1.28–1.45) in patients with GERD than in those without GERD. After stratified analyses for sex, age, and comorbidity, patients with GERD had a higher risk of periodontitis for age (aHR = 1.31, 95% CI = 1.20–1.42 for 40–54 years and aHR = 1.42, 95% CI =1.28–1.57 for 55–69 years), sex (aHR = 1.40, 95% CI = 1.28–1.54 for men and aHR = 1.33, 95% CI = 1.23–1.45 for women), and presence (aHR = 1.36, 95% CI = 1.27–1.45) and absence (aHR = 1.40, 95% CI = 1.21–1.62) of comorbidity than those without GERD. Among the GERD cohort, the risk for periodontitis was increased with an increasing number of emergency room visits (≥ 1 vs. <1, aHR = 5.19, 95% CI = 2.16–12.5). Conclusions: Our results revealed that patients with GERD have a higher risk of periodontitis development than those without GERD. Clinicians should pay more attention to identifying and managing periodontitis in patients with GERD.

## 1. Introduction

Gastroesophageal reflux disease (GERD) occurs when excessive reflux of gastric contents into the esophagus causes troubling symptoms, such as acid regurgitation and heartburn, and long-term reflux may cause severe clinical symptoms such as peptic strictures, esophagitis, and Barrett esophagus [1]. It is the most common esophageal disease worldwide that affects approximately 20% of the adult populations of Western countries. The prevalence of typical GERD-associated symptoms in North America and Europe ranges from 10% to 30%, is lower (~7.8%) in East Asia, and is growing considering changes in modern lifestyle factors, diet and nutrition habits, and genetic predisposition [2]. In the esophagus, reflux of gastric contents may contribute to chronic inflammation, histological changes in the mucosal cells of the distal esophagus, and higher chances of esophageal adenocarcinoma [3,4]. In addition to well-reported clinical manifestations of GERD such as chronic cough, vomiting, chest pain, respiratory diseases, and poor weight gain, dental erosion and teeth loss may also occur [5,6].

Periodontitis is one of the most common chronic inflammatory oral diseases globally that can cause progressive loss of gums and tooth connective tissues, periodontal ligament, and alveolar bone [7]. Earlier epidemiological studies reported that chronic periodontitis more commonly occurs and is more severe in American and European adults than in East Asian adults, with at least 46% of 30-year-olds having periodontitis, and among them, 10% having severe periodontitis [8]. An appropriate cure for periodontitis is essential for maintaining an adequate oral-related health quality of life, and an untreated periodontal condition jeopardizes the intact dentition and can lead to tooth loss, pain, impaired oral function, and malnutrition [9,10]. Periodontitis plaque can also provoke both local and systemic inflammations and is reported to be associated with various systemic diseases, such as cardiovascular diseases [11], respiratory diseases [12], diabetes mellitus [13], rheumatoid arthritis [14], osteoporosis [15], dementia [16], gastric diseases [17,18], and gastric *Helicobacter pylori* infection [19]. On the other hand, systemic diseases might affect the development and progression of periodontitis; thus, exploring possible risk factors for periodontitis is imperative.

Only a few studies have described the possible correlation between GERD and increased risk of periodontitis. Specifically, Dzhamaldinova et al. [20] conducted the first study on the association of periodontitis risk with GERD. A pathogenic link was observed between chronic inflammatory periodontal disease (CIPD) and GERD and was marked by a combined treatment than with isolated treatment for CIPD. Song et al. [21] conducted a small population-based clinical study, reported a significantly higher risk of chronic periodontitis in patients with GERD, and considered it an independent risk factor. Recently, Lee et al. [22] conducted a small population-based clinical study on obstetric cases and showed that GERD closely correlates with periodontitis risk during pregnancy and systemic inflammation-associated preterm birth. However, these studies were based on relatively small samples and were designed as case–control or cross-sectional investigations. To address these limitations, the aim of this study was to conduct a nationwide retrospective cohort study using Taiwan’s National Health Insurance Research Database (NHIRD), which is a medical claims database, to evaluate whether GERD is associated with the development of periodontitis.

## 2. Materials and Methods

### 2.1. Data Sources

A single-payer National Health Insurance (NHI) program was established by the Taiwan Ministry of Health and Welfare in 1995, and it contains universal and comprehensive health claims data of more than 99.5% of the Taiwanese population. The NHIRD was set up and updated by Taiwan Ministry of Health and Welfare.

In this study, we extracted data from a subset of NHIRD, known as the Longitudinal Health Insurance Database (LHID). The LHID includes detailed medical claims information of one million randomly selected people from the NHI program and provides de-identified health care information of individuals regarding demographic characteristics (age, sex, and date of birth and death), medications, diagnostic codes, and procedure and surgery claims from 2008 to 2018. All diagnoses were recorded according to the International Classification of Diseases, Ninth Revision, Clinical Modification (ICD-9-CM).

This study was approved with the permission of the clinical review and research ethics committee of the China Medical University and Hospital (CMUH-107-REC2-181). Informed consent was waved due to whole data extracted from the LHID, which included only comprehensive de-identified healthcare information.

### 2.2. Sample Participants

We used two cohorts for this study: a GERD cohort (case group) and a non-GERD cohort (comparison group). In the GERD cohort (n = 20,125, minimum age = 40 years), participants with newly diagnosed GERD (ICD-9-CM codes 530.11 and 530.81) were identified between 2008 and 2018. The date of diagnosis was defined as the index date. Individuals aged <40 years, had incomplete demographic information, and diagnosed with periodontitis (ICD-9-CM codes 523.3 and 523.4) before the index date were excluded from the GERD cohort. The non-GERD cohort was propensity-matched at a 1:1 ratio with the GERD cohort by age, sex, index year, and comorbidities. The same exclusion criteria were applied for the comparison group.

### 2.3. Study Outcomes and Comorbidities

The primary outcome of interest in this study was the development of periodontitis. All participants of the two groups were followed-up from the index date until periodontitis occurrence and stopped when they withdrew from the NHI program, the end of 2018, or death. Comorbidities associated with periodontitis risk were recorded before the index date. The following comorbidities were included in this study: hypertension (ICD-9-CM codes 401–405), diabetes mellitus (ICD-9-CM code 250), hyperlipidemia (ICD-9-CM code 272), asthma and chronic obstructive pulmonary disease (asthma/COPD, ICD-9-CM codes 491, 492, 493, and 496), chronic liver disease and cirrhosis (CLD, ICD-9-CM code 571), and chronic kidney disease (CKD, ICD-9-CM code 585).

### 2.4. Statistical Analysis

A Chi-squared test was used for age group, sex, and comorbidity, whereas a two-tailed t-test was performed for mean age. To examine the cumulative incidence rate (IR) of periodontitis in both groups, the Kaplan–Meier model was used, and intergroup differences were analyzed using a log-rank test. Furthermore, univariate and multivariate Cox proportional hazardous models were performed to estimate the crude and adjusted hazard ratios (cHRs and aHRs) and 95% confidence intervals (CIs). All data analyses were conducted using SAS (version 9.4 for Windows, SAS Institute Inc., Cary, NC, USA) and R (version 3.2, R Foundation for Statistical Computing, Vienna, Austria). A two-tailed *p*-value of <0.05 was considered to indicate statistical significance.

## 3. Results

**Figure 1:**
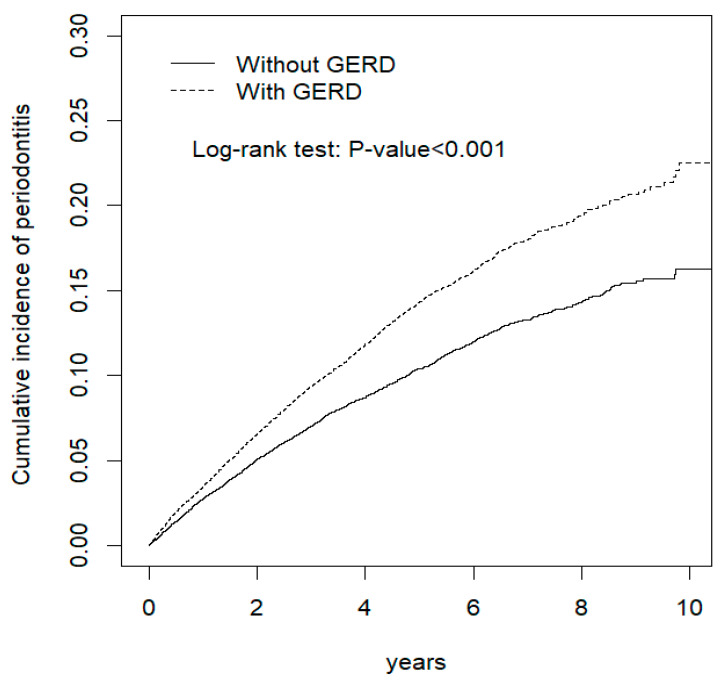
Cumulative incidence of periodontitis between individuals with and without gastroesophageal reflux disease (GERD). Patients with GERD had an increased cumulative risk of periodontitis as compared those without GERD.

We recruited 20,125 patients in both groups (Table 1), of which 53.2% were women, and the median age was 60 years. Both groups showed no significant variations between age, sex, and comorbidities (*p* > 0.05). The cumulative IR of the periodontitis risk between the GERD and non-GERD groups analyzed via a Kaplan–Meier model with a log-rank test is demonstrated in Figure 1. The GERD group exhibited a significantly increased risk of periodontitis than the non-GERD group (log-rank test; *p* < 0.001).

Table 2 represents the overall IRs, cHRs, aHRs, and 95% CIs for periodontitis risk between the groups by GERD, age, and sex. The overall IRs of periodontitis in the GERD group and non-GERD group were 30.0 and 21.7, respectively. Compared with the non-GERD group, the GERD group displayed a significantly increased periodontitis risk even after adjusting for sex, age, and comorbidities (cHR = 1.37, 95% CI = 1.29–1.46 and aHR = 1.36, 95% CI = 1.28–1.45). In addition, the aHRs for the periodontitis risk in all enrolled participants was significantly increased in those aged 40–54 (IR = 28.7, aHR = 1.88, 95% CI = 1.70–2.08) and 55–69 years (IR = 28.7, aHR = 1.83, 95% CI = 1.66–2.03) compared with those aged ≥70 years. No difference was found in periodontitis risk between male and female participants.

Table 3 shows aHRs of periodontitis between the GERD group and the non-GERD group stratified by sex, age, and comorbidity. The aHRs for periodontitis were significantly higher in the GERD group than in the non-GERD group, in those aged 40–54 years (aHR = 1.31, 95% CI = 1.20–1.42), 55–69 years (aHR = 1.42, 95% CI = 1.28–1.57), and ≥70 years (aHR = 1.43, 95% CI = 1.21–1.69); men (aHR = 1.40, 95% CI = 1.28–1.54) and women (aHR = 1.33, 95% CI = 1.23–1.45); patients with (aHR = 1.36, 95% CI = 1.27–1.45) and without (aHR = 1.40, 95% CI = 1.21–1.62) comorbidity.

Table 4 displays that the GERD group had an increased risk for periodontitis development when they visited the emergency room more than once per year (aHR = 5.19, 95% CI = 2.16–12.5) compared with those who visited the emergency room less than once per year.

## 4. Discussion

This study is the first nationwide population-based retrospective cohort study to analyze the occurrence of periodontitis in patients with and without GERD. The GERD group had a significantly increased periodontitis risk compared with the non-GERD group. Furthermore, the aHRs for periodontitis risk in the GERD group were significantly higher in all age groups, sexes, and with the presence or absence of any comorbidity than the non-GERD group. Furthermore, the periodontitis risk proportionally increased in patients with GERD who visited the emergency room more frequently annually.

As already mentioned, few studies have described the association between GERD and periodontitis risk [20,21,22,23]. Some possible mechanisms have postulated the association between GERD and increased periodontitis risk. The pathogenesis of both clinical diseases is more complex and involves numerous cellular mechanisms. These diseases are primarily characterized by systemic inflammation, damaging connective tissues’ barriers to numerous pathogens such as bacteria and toxic substances. They potentially share a similar inflammation-based pathological process, which includes pro-inflammatory cytokines such as interleukins 6, 8, and 9, impaired protease/antiprotease receptor activity, leukocytes, and oxidative stress [24,25,26]. Moreover, some common factors could contribute to the development of both diseases, including modern lifestyle factors (drugs, alcohol, and tobacco smoking), dietary habits, respiratory diseases, and socioeconomic status. Modern lifestyle factors and dietary habits are the most critical confounding factors in this study. However, the NHI program database does not include such types of information. We assume that >50% of the participants had modern lifestyle/dietary habit factors that are highly related to GERD complications in the case group, based on earlier studies [27,28,29]. Our findings also suggest that increased gastric reflux contents may cause poor oral hygiene conditions through poor dietary habits or modern lifestyle factors.

Poor salivary secretion is the most promising reason for GERD as an influencing factor for periodontitis development. Efficient saliva secretion with mucin-rich protective barriers against thermal, mechanical, chemical, and microbial pathogens mediated damage. It also serves as an antiacid to provide endogenous protection against higher gastric acid reflux contents caused by inefficient neutralization of acid [30,31]. Earlier studies reported that hyposalivation in patients with GERD due to impaired esophageal–salivary flow increases the vulnerability of the oral cavity to the acidic saliva (pH 4.5) and proteolytic gastric contents and finally increases the risk of chronic periodontitis in older people [32,33,34] by allowing increased proliferation of pathogenic bacteria and a reduction in the amount of oral self-defense antimicrobial proteins [35,36,37,38,39]. These studies have indicated that GERD may be a predisposing marker for periodontitis risk through abnormal systemic inflammation and hyposalivation. However, the precise explanation of the periodontitis risk in patients with GERD remains unknown. Consistent with these studies, systemic inflammation, hyposalivation, and impaired oral defense mechanisms may play a vital role in the correlation between GERD and periodontitis risk.

This study has several strengths. First, it is a nationwide population-based retrospective cohort study that analyzed the correlation between GERD and periodontitis risk. Second, the accessible clinical data on GERD depended on the NHIRD health claims database reported by specialist clinicians; thus, the study of GERD status was more precise and objective when compared with self-analyzed data. Finally, by using a cohort study design, the GERD status was observed before periodontitis development; therefore, the probable relationship between GERD and periodontitis risk might be more precisely examined.

However, this study has some limitations. First, the current NHIRD did not provide detailed information regarding the participant’s body mass index, modern lifestyle factors, living/dietary habits, family history, oral hygiene practices, socioeconomic status, and environmental factors, which might be possible confounding factors. Second, the health database did not collect clinical variable data such as pro-inflammatory markers, laboratory clinical sample data, detailed dental reports, culture results, records of smoking or alcohol consumption, and dietary habits timing. Third, GERD, periodontitis risk, and comorbidities were diagnosed using ICD codes and guidelines, primarily based on medical professionals. Regular checkups are performed to avoid any negligence and misdiagnoses. Furthermore, periodontal diseases had severity classification, such as mild, moderate, and chronic conditions [40]. The correlation between GERD and stages of periodontitis severity was not examined in this database.

## 5. Conclusions

Taken together, this study shows that patients with GERD may have a higher risk for periodontitis than those without GERD. Clinicians should pay more attention to the development of periodontitis while caring for patients with GERD. On the other hand, dentists may consider GERD as an etiology of unexplained periodontitis. The combination of GERD and periodontitis may further worsen our overall health; more studies should be performed on the issue.

## Figures and Tables

**Table 1:** Demographic characteristics and comorbidities for individuals with and without GERD.

Variables	GERD				*p*-Value ^#^
	Yes		No		
	N = 20,125		N = 20,125		
	n	%	n	%	
Age					0.99
40–54	8335	41.4	8338	41.4	
55–69	6633	33.0	6671	33.2	
≥70	5157	25.6	5116	25.4	
Mean ± SD	60.2	±13.1	60.3	±13.2	0.72
Gender					0.96
Women	10,700	53.2	10,695	53.1	
Men	9425	46.8	9430	46.9	
Comorbidity					
Hypertension	10,910	54.2	10,876	54.0	0.73
Diabetes mellitus	2838	14.1	2831	14.1	0.92
Hyperlipidemia	8690	43.2	8689	43.2	0.99
Asthma/COPD	7498	37.3	7527	37.4	0.77
Chronic liver disease	8803	43.7	8819	43.8	0.87
Chronic kidney disease	1203	5.98	1181	5.87	0.64

COPD, chronic obstructive pulmonary disease; GERD, gastroesophageal reflux disease; SD, standard deviation. ^#^ Chi-squared test and *t*-test.

**Table 2:** Incidence rates and hazard ratios of periodontitis for all participants.

	Event	PY	IR ^#^	Crude HR (95% CI)	Adjusted HR ^‡^ (95% CI)
GERD					
No	1818	83,818	21.7	1.00	1.00
Yes	2361	78,698	30.0	1.37 (1.29–1.46) ***	1.36 (1.28–1.45) ***
Age					
40–54	2082	72,480	28.7	1.93 (1.76–2.12) ***	1.88 (1.70–2.08) ***
55–69	1543	53,774	28.7	1.91 (1.73–2.10) ***	1.83 (1.66–2.03) ***
≥70	554	36,262	15.3	1.00	1.00
Gender					
Women	2252	87,649	25.7	1.00	1.00
Men	1927	74,868	25.7	1.00 (0.94–1.07)	-

CI, confidence interval; GERD, gastroesophageal reflux disease; HR, hazard ratio; IR, incidence rate; PY, person years. ^#^ IR per 1000 person years. ^‡^ Multivariable analysis including age, gender, and comorbidities of hypertension, diabetes mellitus, hyperlipidemia, asthma/chronic obstructive pulmonary disease, chronic liver disease, and chronic kidney disease. *** *p* < 0.001.

**Table 3:** Incidence rates and hazard ratios of periodontitis for individuals with and without GERD.

Variables	GERD						Crude HR (95% CI)	Adjusted HR ^‡^ (95% CI)
	No			Yes				
	Event	PY	IR ^#^	Event	PY	IR ^#^		
Age								
40–54	921	36,978	24.9	1161	35,502	32.7	1.31 (1.20–1.42) ***	1.31 (1.20–1.42) ***
55–69	656	27,667	23.7	887	26,107	34.0	1.42 (1.28–1.57) ***	1.42 (1.28–1.57) ***
≥70	241	19,173	12.6	313	17,089	18.3	1.44 (1.22–1.71) ***	1.43 (1.21–1.69) ***
Gender								
Women	986	44,891	22.0	1266	42,757	29.6	1.34 (1.23–1.46) ***	1.33 (1.23–1.45) ***
Men	832	38,927	21.4	1095	35,941	30.5	1.41 (1.29–1.55) ***	1.40 (1.28–1.54) ***
Comorbidity ^§^								
No	321	15,206	21.1	428	14,363	29.8	1.41 (1.22–1.62) ***	1.40 (1.21–1.62) ***
Yes	1497	68,612	21.8	1933	64,335	30.1	1.37 (1.28–1.46) ***	1.36 (1.27–1.45) ***

CI, confidence interval; GERD, gastroesophageal reflux disease; HR, hazard ratio; IR, incidence rate; PY, person years. ^#^ IR per 1000 person years. ^‡^ Multivariable analysis including age, gender, and comorbidities of hypertension, diabetes mellitus, hyperlipidemia, asthma/chronic obstructive pulmonary disease, chronic liver disease, and chronic kidney disease. ^§^ Individuals with any comorbidity of hypertension, diabetes mellitus, hyperlipidemia, asthma/chronic obstructive pulmonary disease, chronic liver disease, and chronic kidney disease were classified into the comorbidity group. *** *p* < 0.001.

**Table 4:** Incidence rates and hazard ratios of periodontitis for individuals with GERD by mean number of annual emergency room visits.

	Event	IR ^#^	Crude HR (95% CI)	Adjusted HR ^‡^ (95% CI)
Annual emergency room visits				
<1	2356	30.0	1.00	1.00
≥1	5	157.2	4.47 (1.86–10.7) ***	5.19 (2.16–12.5) ***

CI, confidence interval; GERD, gastroesophageal reflux disease; HR, hazard ratio; IR, incidence rate. ^#^ IR per 1000 person years. ^‡^ Multivariable analysis including age, gender and the comorbidities of hypertension, diabetes mellitus, hyperlipidemia, asthma/chronic obstructive pulmonary disease, chronic liver disease, and chronic kidney disease. *** *p* < 0.001.

## Data Availability

All data generated or analyzed during this study are included in this manuscript.
